# Mechanism of the Formation of Electronically Excited Species by Oxidative Metabolic Processes: Role of Reactive Oxygen Species

**DOI:** 10.3390/biom9070258

**Published:** 2019-07-05

**Authors:** Pavel Pospíšil, Ankush Prasad, Marek Rác

**Affiliations:** Department of Biophysics, Centre of the Region Haná for Biotechnological and Agricultural Research, Faculty of Science, Palacký University, Šlechtitelů 27, 783 71 Olomouc, Czech Republic

**Keywords:** electronically excited species, reactive oxygen species, oxidative radical reactions, superoxide anion radical, hydrogen peroxide, hydroxyl radical, singlet oxygen, chromophores

## Abstract

It is well known that biological systems, such as microorganisms, plants, and animals, including human beings, form spontaneous electronically excited species through oxidative metabolic processes. Though the mechanism responsible for the formation of electronically excited species is still not clearly understood, several lines of evidence suggest that reactive oxygen species (ROS) are involved in the formation of electronically excited species. This review attempts to describe the role of ROS in the formation of electronically excited species during oxidative metabolic processes. Briefly, the oxidation of biomolecules, such as lipids, proteins, and nucleic acids by ROS initiates a cascade of reactions that leads to the formation of triplet excited carbonyls formed by the decomposition of cyclic (1,2-dioxetane) and linear (tetroxide) high-energy intermediates. When chromophores are in proximity to triplet excited carbonyls, the triplet-singlet and triplet-triplet energy transfers from triplet excited carbonyls to chromophores result in the formation of singlet and triplet excited chromophores, respectively. Alternatively, when molecular oxygen is present, the triplet-singlet energy transfer from triplet excited carbonyls to molecular oxygen initiates the formation of singlet oxygen. Understanding the mechanism of the formation of electronically excited species allows us to use electronically excited species as a marker for oxidative metabolic processes in cells.

## 1. Introduction

Oxidative metabolic processes, such as cellular respiration or oxidative burst, are associated with the formation of reactive oxygen and nitrogen species, which are known to play an important role in the defense against infection, cell signaling, apoptosis, and ageing [1,2,3,4,5,6,7]. Their mutual reactions lead to the formation of other bioactive molecules, such as peroxynitrite [6,8]. For the sake of simplicity, only the reactions with reactive oxygen species (ROS) are reviewed here. It is well established that ROS are formed either by electron transport from highly reducing species to molecular oxygen or by excitation energy transfer from triplet chromophores to molecular oxygen [1]. The electron transport from highly reducing compounds to molecular oxygen results in the formation of a superoxide anion radical (O_2_^•-^), which is known to be further reduced to hydrogen peroxide (H_2_O_2_) and hydroxyl radical (HO^•^). The triplet-singlet energy transfer from the triplet chromophores to molecular oxygen forms singlet oxygen (^1^O_2_). Whereas, the non-radical ROS (H_2_O_2_) are rather less reactive, the radical ROS (HO^•^) are considered to be highly reactive, mainly due to the presence of unpaired electrons and high positive redox potential [1]. It is well established that the radical ROS have a capability to oxidize various biomolecules, such as lipids, proteins, and nucleic acids in the cell [1,9].

The deleterious effect of ROS on biomolecules is prevented by the action of the non-enzymatic and enzymatic antioxidant defense systems. The non-enzymatic antioxidant systems comprise low-molecular weight metabolites (ascorbate, α-tocopherol, carotenoids, glutathione, coenzyme Q10, bilirubin, melanin, lipoic acid, trans-resveratrol), whereas enzymatic scavenging is maintained by enzymes, such as the superoxide dismutase (SOD) family of enzymes (Fe-SOD, Mn-SOD, Ni-SOD, and Cu-Zn SOD) and the peroxidase family of enzymes (ascorbate peroxidase, cytochrome c peroxidase, glutathione peroxidase, and catalase) [10,11,12,13,14,15,16]. However, when the formation of ROS exceeds the capacity of antioxidant systems, the oxidation of polyunsaturated fatty acids, amino acids, and nucleic acids by the deleterious ROS leads to the damage of cellular compartments and, finally, cellular death [17,18].

As a by-product of oxidative metabolic processes, electronically excited species, such as triplet excited carbonyl (^3^R=O^*^) are formed by the decomposition of cyclic (1,2-dioxetane) and linear (tetroxide) high-energy intermediates [19]. The triplet-triplet energy transfer from ^3^R=O^*^ to chromophores initiates the formation of singlet excited (^1^C^*^) and triplet excited (^3^C^*^) chromophores, whereas the triplet-singlet energy transfer from ^3^R=O^*^ to molecular oxygen forms ^1^O_2_ [20]. The electronic transition from the excited state to the ground state is accompanied by ultra-weak photon emission. Several lines of evidence indicate that ultra-weak photon emission is an intrinsic property of all biological systems, including microorganisms, plants, and animals [21,22,23,24,25,26,27]. The intensity of ultra-weak photon emission is considerably low and is generally considered to be within the order of several units or tens photons s^−1^ cm^−2^ [28]. When biological systems are subjected to various biotic (herbivores and pathogens) or abiotic (adverse environmental conditions) stresses, the intensity of ultra-weak photon emission is markedly enhanced to several hundred photons s^−1^ cm^−2^ or more [29,30,31,32,33,34,35,36,37]. The spectral distribution of ultra-weak photon emission is considered in the spectral range from the near ultra-violet A (UVA), to the visible and infra-red (IR) regions of the spectrum [38,39,40]. It is generally considered that ^3^R=O^*^ contributes to the photon emission in the near UVA and blue-green regions of the spectrum, whereas the ultra-weak photon emission of ^1^C^*^ and ^3^C^*^ is in the green-red and near IR region of the spectrum, respectively. The dimol and monomol photon emissions of ^1^O_2_ are in the red and near IR regions of the spectrum, respectively [38,41,42].

Bringing a detailed understanding of the mechanisms of the formation of electronically excited species permits the use of ultra-weak photon emission as a diagnostic tool in basic and applied research. In basic research, electronically excited species can be used as a marker of lipid peroxidation, protein oxidation, and nucleic acid oxidation during the oxidative metabolic processes in varied living systems, ranging from simple unicellular organisms to complex animal systems. In the applied research, the detection of electronically excited species bears the potential to pave way for its application in diagnosis.

## 2. Role of ROS in the Formation of Electronically Excited Species

### 2.1. Superoxide Anion Radical

#### 2.1.1. Formation

**Figure 1:**
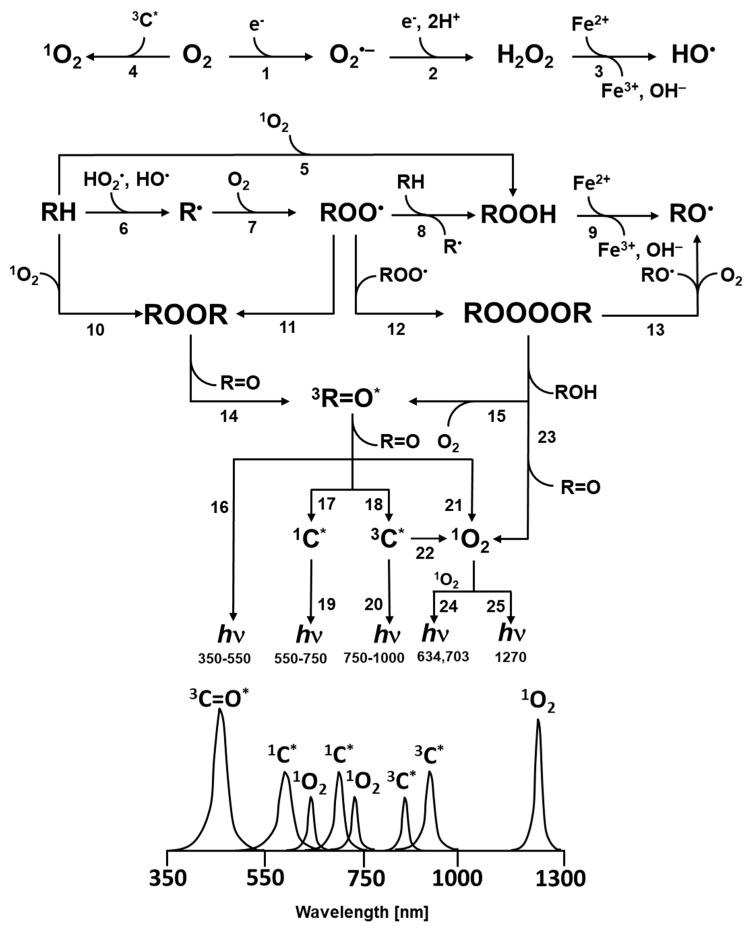
Mechanism of the formation of electronically excited species by oxidative metabolic processes. One-electron reduction of molecular oxygen by highly reducing species forms O_2_^•-^ (reaction 1). The dismutation of O_2_^•-^ generates H_2_O_2_ (reaction 2), whereas the subsequent one-electron reduction of H_2_O_2_ leads to the formation of HO^•^ (reaction 3). The triplet-triplet energy transfer from triplet chromophore to molecular oxygen results in the formation of ^1^O_2_ (reaction 4). Formation of ROOH from R and ^1^O_2_ via ene reaction (reaction 5). The hydrogen abstraction from biomolecules (lipids, proteins and nucleic acids) (RH) by radical ROS (HO^•^, HO_2_^•^) generates R^•^ (reaction 6). The subsequent one-electron oxidation of R^•^ brings about the formation ROO^•^ (reaction 7). The consequent hydrogen abstraction from another R by ROO^•^ forms ROOH (reaction 8). The one-electron reduction of ROOH results in the formation of RO^•^ and OH^−^ (reaction 9). ROOR is formed by either the cycloaddition of ^1^O_2_ (reaction 10) or the cyclization of ROO^•^ (reaction 11). ROOOOR is formed by the recombination of two ROO^•^ (reaction 12). The decomposition of ROOR (reaction 14) or ROOOOR (reaction 15) results in the formation of ^3^R=O^*^. Alternatively, the decomposition of ROOOOR can lead to the formation of two RO^•^ and O_2_ (reaction 13). The electronic transition from ^3^R=O^*^ to R=O is accompanied by the photon emission (16). The energy transfer from ^3^R=O^*^ to chromophores results in the formation of ^1^C^*^ (reaction 17) and ^3^C^*^ (reaction 18) chromophores. The electronic transition from ^1^C^*^ and ^3^C^*^ to the ground state of chromophores is accompanied by the photon emission. (reaction 19, 20). The triplet-triplet energy transfer from ^3^R=O^*^ (reaction 21) and ^3^C^*^ (reaction 22) to molecular oxygen forms (^1^O_2_). Alternatively, the decomposition of ROOOOR via the Russell-type mechanism, results in ^1^O_2_ (reaction 23) with dimol photon emission (reaction 24) and monomol photon emission (reaction 25).

**Figure 2:**
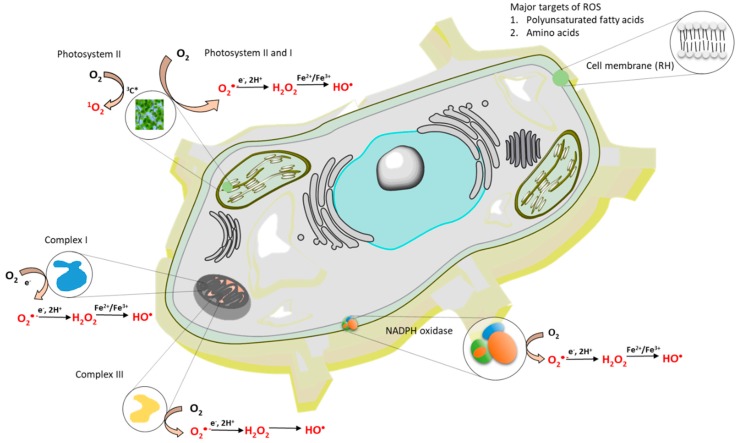
The formation of reactive oxygen species (ROS) within a cell. The formation of ROS occurs in mitochondria, chloroplasts and cell membrane.

**Figure 3:**
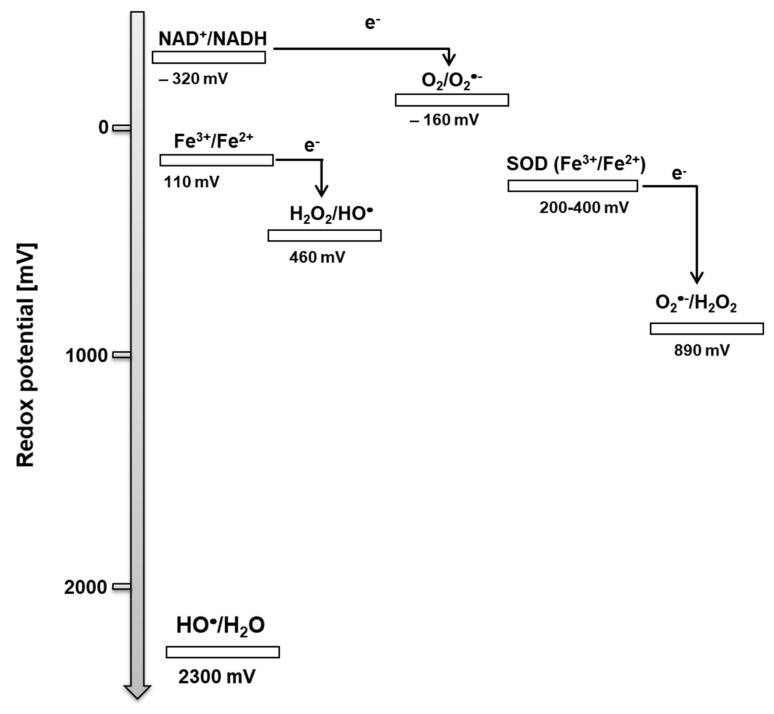
The oxidative properties of ROS. One-electron standard (*E*’_0_) and midpoint (*E*m) redox potentials (pH 7) for redox couples involved in the formation of ROS.

Superoxide anion radical is formed by the one-electron reduction of molecular oxygen (Figure 1, reaction 1). The one-electron reduction of molecular oxygen occurs in the mitochondria during cellular respiration, in the chloroplast during the light reaction of photosynthesis and in the membrane-bound enzyme complex NADPH oxidase during the oxidative burst. [43,44] (Figure 2). The superoxide anion radical in the mitochondria is known to be formed by complex I and complex III [43]. In chloroplasts, molecular oxygen is reduced by the highly reducing cofactors on the acceptor side of both photosystem II [45] and photosystem I [46]. The one-electron reduction of molecular oxygen during the oxidative burst catalyzed by the NADPH oxidase occurs in the plasma membrane of phagocytic cells, such as neutrophils, eosinophils, monocytes, and macrophages [47,48]. Because the standard redox potential of O_2_/O_2_^•-^ redox couple is −160 mV (pH 7) [49] (Figure 3), the redox components with a high negative midpoint redox potential have the capability to reduce molecular oxygen.

#### 2.1.2. Oxidizing Property

It has been previously demonstrated that under normal conditions, O_2_^•-^ is unable to oxidize polyunsaturated fatty acids, amino acids, and nucleic acids [50,51]. Based on these observations, it is generally considered that the reactivity of O_2_^•-^ toward polyunsaturated fatty acids, amino acids, and nucleic acids is rather limited. A few pieces of evidence suggest that O_2_^•-^ has the capability to oxidize an iron-sulphur cluster or interact with a radical, such as the tyrosine phenoxyl radical, which is formed by hydrogen abstraction from the hydroxyl group of tyrosine [52]. Although O_2_^•-^ is relatively less reactive compared to other ROS, the potential deleterious effect of O_2_^•-^ is due to its ability to mediate the reduction of H_2_O_2_ into highly oxidizing radicals, such as HO^•^. Compared with the anionic form of the O_2_^•-^, the protonated form of the superoxide radical, known as the perhydroxyl radical (HO_2_^•^), is considered to be more reactive. The formation of HO_2_^•^ (pKa 4.8) [50] occurs particularly at the surface of the membrane, where the concentration of protons is high. Evidence has shown that HO_2_^•^ is an oxidizing agent that can directly abstract hydrogen from polyunsaturated fatty acids, amino acids, and nucleic acids [50,51]. The increased ability of HO_2_^•^ to abstract hydrogen from polyunsaturated fatty acids, amino acids, and nucleic acids is due to the greater oxidizing ability (*E*_0′_ of O_2_^•-^/H_2_O_2_ and HO_2_^•^/H_2_O_2_ redox couple is 0.89 V and 1.06 V, respectively) and the lack of a negative charge on the molecule [53]. It has been proposed that polyunsaturated fatty acids, amino acids, and nucleic acids are oxidized at the site of O_2_^•-^ production, whereas HO_2_^•^ oxidizes polyunsaturated fatty acids, amino acids, and nucleic acids at a greater distance from the site of its production, due to its higher mobility.

### 2.2. Hydrogen Peroxide

#### 2.2.1. Formation

Hydrogen peroxide is formed by either the one-electron reduction of O_2_^•-^ (Figure 1, reaction 2) [54,55]. One-electron reduction of O_2_^•-^ either occurs spontaneously or is catalyzed by various types of SOD [44]. There are three major families of SOD depending on the metal cofactor: Cu/Zn, Fe, Mn, and the Ni type. Cu/Zn SOD is most commonly used by eukaryotes. Fe and Mn SOD can be found in mitochondria and prokaryotes, together with Ni SOD. As two molecules with the same charge are repulsed, the interaction of two negatively charged O_2_^•-^ is rather limited. By contrast, due to the lack of charge on the protonated form of superoxide HO_2_^•^, HO_2_^•^ easily interacts either with the negatively charged O_2_^•-^ or the neutral HO_2_^•^. Because the pKa value of HO_2_^•^ is 4.8 [50], HO_2_^•^ is formed in an acidic microenvironment, whereas O_2_^•-^ is preferentially formed at the neutral pH. When a high concentration of protons is available with the medium (low pH), the spontaneous dismutation is favored, whereas at the physiological pH, the dismutation reaction is preferably catalyzed by SOD. The enzymatic dismutation of O_2_^•-^ comprises two reactions: The one-electron oxidation of O_2_^•-^ to molecular oxygen (superoxide oxidase) and the one-electron reduction of O_2_^•-^ to H_2_O_2_ (superoxide reductase) [44]. Because the standard redox potential of the O_2_/O_2_^•-^ redox couple is −160 mV (pH 7) [56] and the standard redox potential of the O_2_^•-^/H_2_O_2_ redox couple is 890 mV (pH 7) [49], the redox active metal of SOD must be in the range of −160 mV to 890 mV. Evidence has shown that the midpoint redox potential of the redox active centre in SOD ranges from 200 to 400 mV, depending on the type of the metal active centre [57] (Figure 3). The one-electron reduction of O_2_^•-^ catalyzed by SOD occurs predominantly in the mitochondria, peroxisomes, and cytoplasm. In addition to one-electron reduction, the two-electron reduction of molecular oxygen is catalyzed by various types of oxidases, such as monoamine oxidase in mitochondria, urate oxidase, L-alpha-hydroxy acid oxidase, polyamine oxidase, oxalate oxidase, and fatty acyl- CoA oxidase in peroxisomes [1,58] (Figure 2). Further, the two-electron oxidation of H_2_O by the water splitting manganese complex in photosystem II in chloroplasts can lead to the formation of H_2_O_2_ [59].

#### 2.2.2. Oxidizing Property

It is well established that H_2_O_2_ is poorly reactive, with almost no capability to oxidize polyunsaturated fatty acids and nucleic acids, and limited capability to oxidize amino acids, [58,60]. Evidence has shown that of all amino acids, few are oxidized by H_2_O_2_, including cysteine, tryptophan, tyrosine, histidine, and methionine [61,62]. The oxidation of cysteine by H_2_O_2_ has been reported to form cysteine sulphenic acid, while a sulphurane derivative is formed upon the oxidation of cysteine, conjugated with histidine residue [63]. Though H_2_O_2_ is rather unreactive compared with other ROS, the potentially deleterious effect of H_2_O_2_ is due to its ability to serve as a substrate for highly oxidizing radicals, such as HO^•^ [53]. It is generally accepted that H_2_O_2_ might cause deleterious effect polyunsaturated fatty acids, amino acids, and nucleic acids far from the production site. 

### 2.3. Hydroxyl Radical

#### 2.3.1. Formation

The hydroxyl radical is formed through the one-electron reduction of H_2_O_2_ (Figure 1, reaction 3). For a long time, the production of HO^•^ in biological systems was believed to occur by Fenton reaction, i.e., the reduction of free H_2_O_2_, mediated by free metal ions, such as Fe^2+^, Mn^2+^, Cu^+^, or Zn^+^ [64,65] (Figure 2). From the thermodynamic point of view, the reduction of H_2_O_2_ by free ferrous iron is feasible because the standard redox potential of the ferric/ferrous redox couple [(*E*_0_’(Fe^3+^/Fe^2+^) = 110 mV, pH 7] [66] is lower than the standard redox potential of the H_2_O_2_/HO^•^ redox couple [*E*_0_’(H_2_O_2_/HO^•^) = 460 mV, pH 7] [67] (Figure 3). In biological systems, iron is stored in the ubiquitous protein, ferritin, which is known to receive several thousand iron atoms [68]. Because the solubility of iron is low at physiological pH, chelators, such as reduced flavins, cysteine, and glutathione, maintain the transport of iron from ferritin to other biomolecules [69]. Because the concentration of iron in biological systems is low, the re-reduction of ferric to ferrous iron is required to carry out the reduction of H_2_O_2_ to HO^•^ in numerous reactions. The restoration of ferrous iron is maintained by reducing compounds, such as O_2_^•-^. It is currently accepted that the superoxide-driven Fenton reaction, often called the Haber–Weiss reaction, is primarily responsible for the formation of HO^•^ in biological systems [70]. Apart from free H_2_O_2_, evidence has shown that HO^•^ is formed by the reduction of peroxide coordinated to the metal center via the ferric-oxo species as an intermediate product [71].

#### 2.3.2. Oxidizing Property

Due to the highly positive redox potential of the HO^•^/H_2_O redox couple [*E*_0_’(HO^•^/H_2_O) = 2.3 V, pH 7] [66], HO^•^ is highly reactive towards polyunsaturated fatty acids, amino acids, and nucleic acids. In polyunsaturated fatty acids, the hydrogen abstraction occurs primarily from the carbon next to the double bond, leading to the formation of lipid alkyl radicals (R^•^). Similarly, in the amino acids, the initiation of oxidation by HO^•^ occurs through the abstraction of the H-atom at the α-carbon [72]. Hydrogen abstraction by HO^•^ has been reported for aliphatic amino acids, such as glycine, alanine, valine, leucine, isoleucine, and proline, while oxidation by the addition of HO^•^ has been reported for aromatic amino acids, such as phenylalanine, tryptophan. and tyrosine [61]. Hydrogen abstraction from polyunsaturated fatty acids and amino acids by HO^•^ leads to the formation of lipid and protein alkyl radicals (R^•^). The formation of R^•^ from polyunsaturated fatty acids and amino acids by HO^•^ initiates a cascade of reactions leading to lipid peroxidation and protein oxidation. The oxidation of DNA by HO^•^ has been reported to occur in two different ways [73,74,75]. The first way comprises a strand break due to the oxidation of sugars, whereas the second way involves alternating the bases. Sugar damage is initiated by hydrogen abstraction from one of the deoxyribose carbons. Oxidation of the bases results primarily in HO^•^ being added to the electron-rich double bounds, such as guanine forming 8-hydroguanine [76,77]. It is generally accepted that HO^•^ reacts with polyunsaturated fatty acids, amino acids, and nucleic acids in the proximity of the production site, with limited diffusion to other targets far from the production site. 

### 2.4. Singlet Oxygen 

#### 2.4.1. Formation

Singlet oxygen is formed by the triplet-singlet energy transfer from ^3^C^*^ to molecular oxygen (Figure 1, reaction 4) [78]. In this reaction, the absorption of UV radiation or visible light by various types of natural chromophores, including tetrapyrroles (porphyrin, bilirubin), flavin (FMN, RAD), pyridine nucleotides (NADH, NADPH), melanin, urocanic acid, and pterins, forms ^1^C^*^ subsequently transformed into ^3^C^*^ through intersystem crossing [79]. The triplet energy of chromophores is transferred to molecular oxygen forming ^1^O_2_, while the chromophores return to the ground state. The excitation energy transfer from ^3^C^*^ to molecular oxygen occurs to either the first singlet excited state (¹Δ_g_) or the second singlet excited state (¹Σ_g_^+^). The first singlet excited state (¹Δ_g_), which has two paired electrons in the same molecular orbital, has an energy level of 22.5 kcal mol^−1^ [80]. The second singlet excited state (¹Σ_g_^+^), which has two electrons in different orbitals with antiparallel spin, has an energy level of 31.5 kcal mol^−1^ [81]. From the thermodynamic point of view, the triplet-singlet energy transfer from ^3^C^*^ to both the first singlet excited state (¹Δ_g_) and the second singlet excited state (¹Σ_g_^+^) is feasible, because the energy level of the singlet excited state (¹Δ_g_ or ¹Σ_g_^+^) is below the triplet energy level of the chromophores (the activation energy of ^3^C^*^ varies from 28 kcal/mol to 38 kcal/mol). 

#### 2.4.2. Oxidizing Property

Singlet oxygen does not abstract hydrogen from polyunsaturated fatty acids, amino acids, or nucleic acids, but it can react with biomolecules to form lipid, protein, and nucleic acid endoperoxides and hydroperoxides (ROOH) [82,83]. Endoperoxides formed by the cycloaddition of ^1^O_2_ to polyunsaturated fatty acids and amino acids are known to decompose to non-radical products, such as malondialdehyde (MDA) [84]. Hydroperoxides formed by ^1^O_2_ through the ene reaction with polyunsaturated fatty acids, amino acids, and nucleic acids [85,86] are oxidized and reduced to lipid, protein, and DNA peroxyl (ROO^•^) and alkoxyl (RO^•^) radicals, respectively. Whereas the endoperoxide decomposition leads to the termination of lipid peroxidation and protein oxidation, the decomposition of ROOH leads to the propagation of oxidizing chain reactions. It has been reported that the oxidation of nucleic acids by ^1^O_2_ results in the alternation of the bases [87,88,89]. A high reactivity of ^1^O_2_ towards guanine has been shown, resulting in the formation of 8-oxo-7,8-dihydro-2-deoxyguanosine [87,90,91]. Time-resolved ^1^O_2_ phosphorescence experiments performed in single cells showed that the intracellular lifetime of ^1^O_2_ is 3 µs [78]. The apparent spatial domain of ^1^O_2_ activity within the cell will likely have a spherical radius of 100 nm [92,93,94]. More detailed information about the oxidizing properties of ^1^O_2_ can be found in another excellent review [95].

## 3. Mechanism of the Formation of Electronically Excited States

### 3.1. Organic Radicals

**Figure 4:**
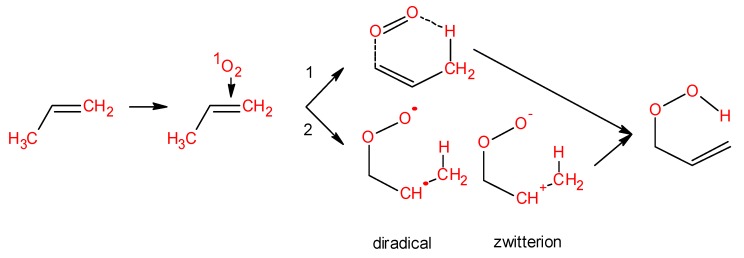
Formation of lipid ROOH via ene reaction. Ene reaction occurs through two different ways: Concerted pathway (reaction 1) and stepwise mechanism (reaction 2). During the stepwise mechanism 2, different intermediates are formed: Diradical and zwitterion.

The abstraction of weakly bonded hydrogen atoms from polyunsaturated fatty acids and amino acids by HO^•^ forms lipid and protein R^•^, respectively (Figure 1, reaction 6). When molecular oxygen is present, R^•^ interacts at a diffusion limited rate with molecular oxygen to form lipid and protein ROO^•^ (Figure 1, reaction 7). The hydrogen abstraction by ROO^•^ from lipids and proteins leads to the formation of lipid and protein ROOH [96] (Figure 1, reaction 8). Alternatively, in the presence of ^1^O_2_ the lipid and protein ROOH are formed via ene reaction (Figure 1, reaction 5) [97]. In this reaction, lipid and protein react with ^1^O_2_ in either a concerted or stepwise mechanism. Concerted pathway comprises the orbital interaction of HOMO of C=C in polyunsaturated fatty acids and amino acids with a LUMO of ^1^O_2_ (Figure 4, reaction 1) [85]. In the stepwise mechanism, lipid and protein ROOH are formed via transitional intermediates, comprising diradical and zwitterion [86] (Figure 4, reaction 2). In spite of the fact that experimental evidence has not been provided, it is proposed here that the formation of DNA ROOH occurs through the above described reaction pathway.

When transition metals and reducing agents are lacking, the lipid and protein ROOH are highly stable and accumulate in the cells [98,99]. When transition metals and reducing agents are present in close proximity to ROOH, ROOH is reduced by transition metals, such as Fe^2+^, Mn^2+^**,** Cu^+^, or Zn^+^ to lipid and protein RO^•^ (Figure 1, reaction 9).

### 3.2. High-Energy Intermediates

The chain reactions of organic radicals result in the formation of cyclic and linear high-energy intermediates. Cyclic high-energy intermediates are predominantly 1,2-dioxetanes (ROOR), whereas linear high-energy intermediates comprise tetroxides (ROOOOR) [100,101]. Several lines of evidence have shown that ROOR are formed either by the cycloaddition of ^1^O_2_ to polyunsaturated fatty acids and amino acids (Figure 1, reaction 10) or by the cyclisation of ROO^•^ (Figure 1, reaction 11) [102]. Tetroxides are formed by the recombination of ROO^•^ (Figure 1, reaction 12).

#### 3.2.1. 1,2-Dioxetane

**Figure 5:**
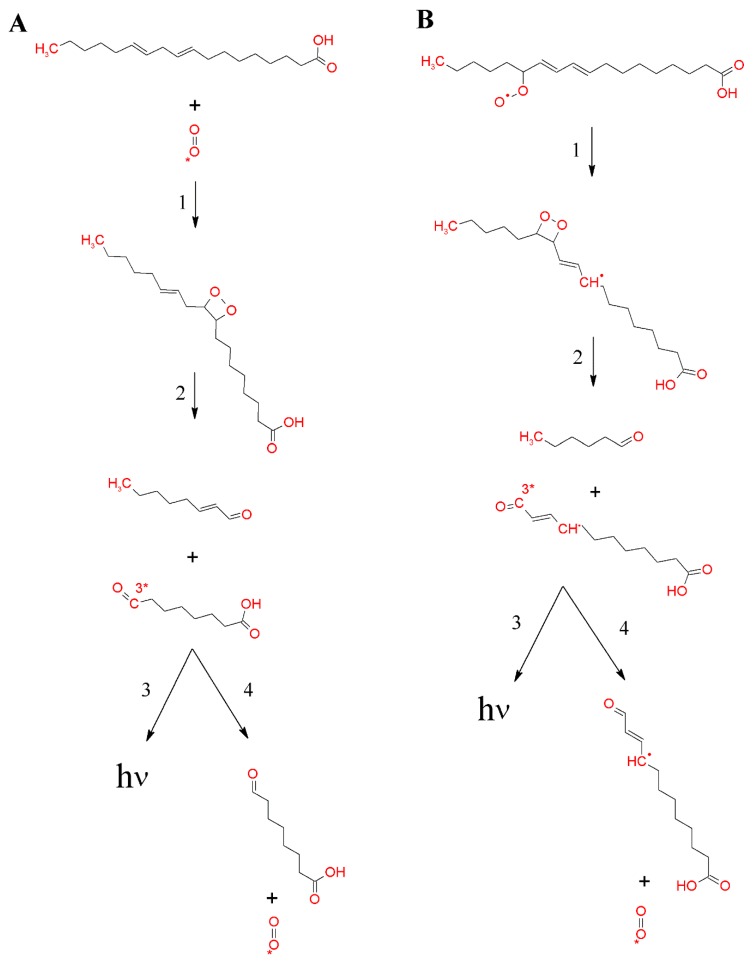
Formation of 1,2-dioxetane by the cycloaddition of ^1^O_2_ to polyunsaturated fatty acid (**A**) or the cyclisation of lipid peroxyl radicals (**B**). Formation of 1,2-dioxetane (reaction 1). 1,2-dioxetane is known to decompose into ^3^R=O^*^ and R=O (reaction 2). Subsequently, the electronic transition from ^3^R=O^*^ to R=O is associated with photon emission (reaction 3), or the energy transfer from ^3^R=O^*^ to molecular oxygen causes the formation of ^1^O_2_ (reaction 4).

Figure 5 shows in detail the formation of ROOR, either by the cycloaddition of ^1^O_2_ to polyunsaturated fatty acids (panel A) or by the cyclisation of ROO^•^ (panel B). The stereospecific ^1^O_2_ [2 + 2] cycloaddition has been shown to form ROOR, consisting of two carbon atoms joined to two oxygen atoms (Figure 5A, reaction 1), whereas stereospecific ^1^O_2_ [4 + 2] cycloaddition has been shown to generate endoperoxide, consisting of four carbon atoms joined to two oxygen atoms [16,100,102,103,104,105]. Evidence has shown that ROOR is formed predominantly in an acidic environment via a protonated intermediate or in a nonpolar environment via an unprotonated intermediate [100]. It seems likely that, at the membrane edge, ROOR is formed via a protonated intermediate, while in the interior membrane, ROOR is formed via an unprotonated intermediate. Alternatively, the cyclisation of ROO^•^ has been shown to form ROOR with R^•^ (Figure 5B, reaction 1). Subsequently, the R^•^ reaction with molecular oxygen, forming another ROO^•^, has been shown to result in the further cyclisation of ROO^•^ to produce bicyclic ROOR [106]. 

#### 3.2.2. Tetroxide

**Figure 6:**
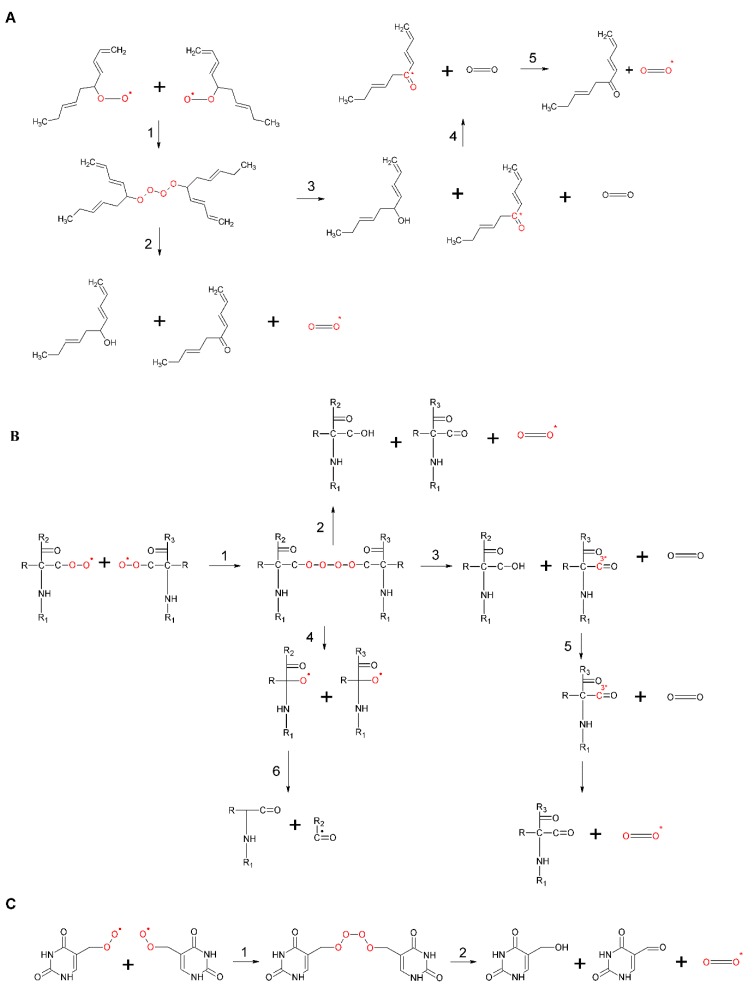
Formation of ROOOOR by the recombination of two lipid ROO^•^ (**A**), protein ROO^•^ (**B**), and DNA ROO^•^ (**C**). In (**A**–**C**), the recombination of two ROO^•^ results in the formation of unstable ROOH (reaction 1). ROOH can decompose either to ground carbonyl, ^1^O_2_ and ROH (reaction 2) or to ^3^R=O^*^, molecular oxygen and ROH (reaction 3). The triplet-singlet energy transfer from ^3^R=O^*^ to molecular oxygen causes the formation of ^1^O_2_ (reactions 4). In (**B**), ROOH can decompose into two RO^•^ in the presence of reducing agents (reaction 5).

The oxidation of lipid, protein, and nucleic acid ROOH by metal ions, haemproteins, peroxynitrite, chloroperoxide, and hypochlorous acid results in the formation of ROO^•^. Apart from a non-radical oxidant, RO^•^ has been proposed to be the cause of the oxidation of ROOH to ROO^•^ [107,108]. The recombination of ROO^•^ initiates the formation of linear ROOOOR via the Russell mechanism (Figure 6, reaction 1) [109,110,111,112]. The recombination of two ROO^•^ into ROOOOR requires that the lifetime of ROO^•^ is sufficient for the formation of a high energy intermediate. To ensure the recombination of two ROO^•^, it seems to be required that the ROO^•^ concentration in the local environment is high enough. It thus seems likely that ROOOOR formation occurs during the termination of lipid peroxidation, protein oxidation, and nucleic acid oxidation. To fulfil all of the conditions mentioned, it is suggested here that, apart from the recombination of two lipid ROO^•^ or two protein ROO^•^, the lipid-protein ROO^•^ cross reaction might occur in biological systems. It has been demonstrated that t-butyl ROOH does not participate in the ROOOOR formation because the presence of α-hydrogen is required to undergo the Russell pathway [113,114]. Based on this observation, it has been established that only the primary and secondary ROO^•^ are involved in the ROOOOR formation, while the tertiary ROO^•^ undergoes propagation of lipid peroxidation and protein oxidation. It has been shown that decomposition of ROOOOR might result in the formation of two RO^•^ (Figure 6B, reaction 4) [3]. 

### 3.3. Electronically Excited States

The decomposition of high-energy intermediates ROOR (Figure 1, reaction 14) and ROOOOR (Figure 1, reaction 15) results in the formation of ^3^R=O^*^ [19,80,115]. In the absence of chromophores or under anaerobic conditions, ^3^R=O^*^ undergoes the electronic transition from the triplet excited state to the ground state, accompanied by photon emission in the near UVA and blue-green regions of the spectrum (350–550 nm) (Figure 1, reaction 16) [116,117]. In the presence of chromophores, the excitation energy is transferred from ^3^R=O^*^ to chromophores [118]. The triplet-singlet and triplet-triplet energy from ^3^R=O^*^ to chromophores forms ^1^C^*^ (Figure 1, reaction 17) and ^3^C^*^ (Figure 1, reaction 18). The excited chromophores undergo the electronic transition from the singlet and triplet excited state to the ground state, accompanied by photon emission in the green-red (550–750 nm) (Figure 1, reaction 19) and the near IR (750–1000 nm) (Figure 1, reaction 20) regions of the spectrum, respectively, depending on the type of chromophores [119]. Under aerobic conditions, the excitation energy is transferred from ^3^R=O^*^ to molecular oxygen [118]. The triplet-singlet energy transfer from ^3^R=O^*^ (Figure 1, reaction 21) and ^3^C^*^ (Figure 1, reaction 22) to molecular oxygen forms ^1^O_2_. Apart from the formation of ^1^O_2_ by triplet-singlet energy transfer from ^3^R=O^*^ to molecular oxygen, ^1^O_2_ is also formed by the decomposition of ROOOOR via a Russell-type mechanism (Figure 1, reaction 23). It has been established that the recombination of ROO^•^ generates 3–14% of ^1^O_2_, where the formation of ^3^R=O^*^ is lower by 3–4 orders of magnitude [120]. Simultaneous photon emission upon collision of two ^1^O_2_ results in dimol photon emission in the red region of the spectrum (634 nm, 703 nm) (Figure 1, reaction 24). Singlet oxygen undergoes the electronic transition from the singlet excited state to the ground triplet state, accompanied by the monomol photon emission in the near IR region of the spectrum at 1270 nm (Figure 1, reaction 25) [121].

#### 3.3.1. Triplet Carbonyl

**Figure 7:**
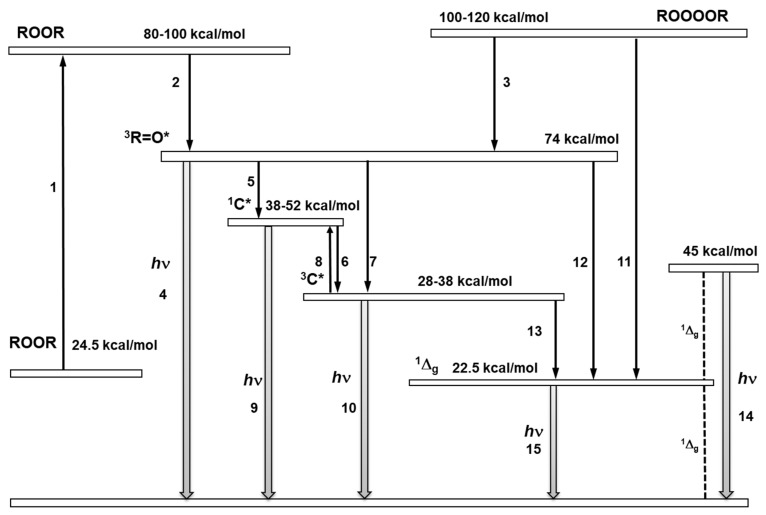
Schematic energy level diagram of the formation of electronically excited species through the decomposition of ROOR and ROOOOR. The decomposition of ROOR occurs via a transition state (reaction 1). The decomposition of the transition state of ROOR generates ^3^R=O^*^ (reaction 2). The decomposition of ROOOOR to ^3^R=O^*^ (reaction 3). The electronic transition from the triplet energy level of ^3^R=O^*^ to the ground state is accompanied by light emission (reaction 4). The triplet-singlet energy transfer from ^3^R=O^*^ to ^1^C^*^ (reaction 5). The triplet-triplet energy transfer from ^3^R=O^*^ to chromophore ^3^C^*^ (reaction 6). Intersystem crossing from ^1^C^*^ to ^3^C^*^ (reaction 6). The reverse intersystem crossing converts ^3^C^*^ to ^1^C^*^ (reaction 8). The electronic transition from ^1^C^*^ and ^3^C^*^ to the ground state is accompanied by photon emission (reaction 9,10). The decomposition of ROOOOR generates singlet oxygen (^1^Δ_g_) (reaction 11). The triplet-singlet energy transfer from ^3^R=O^*^ to molecular oxygen is feasible (reaction 12). The electronic transition from the energy level of singlet oxygen (^1^Δ_g_) to the triplet energy level of ground state is accompanied by dimol (reaction 14) and monomol photon emission (reaction 15).

**Figure 8:**
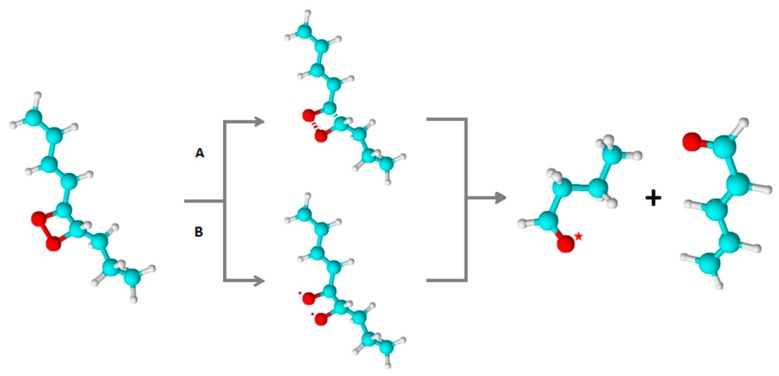
Thermal decomposition of 1,2-dioxetane by concerted (**A**) and diradical (**B**) mechanisms. In (**A**), the concerted mechanism involves the simultaneous cleavage of oxygen-oxygen and carbon-carbon bonds. In (**B**), diradical mechanism contains the cleavage of the oxygen-oxygen bond, resulting in the formation of diradical, followed by the cleavage of the carbon-carbon bond. Both mechanisms result in the formation of triplet exited carbonyl and ground state carbonyl.

The 1,2-dioxetane formed by the cycloaddition of ^1^O_2_ to the polyunsaturated fatty acids and amino acids, or by the cyclisation of ROO^•^, decomposes to triplet excited ^3^R=O^*^ and ground carbonyls (R=O) (Figure 5A and B, reaction 2) [19,122,123,124]. Because the activation energy of ROOR is 24.5 kcal/mol, the decomposition of ROOR to ^3^R=O^*^ (74 kcal/mol) is thermodynamically unfeasible. The energy of 60–80 kcal/mol is needed for the activation of ROOR to its high-energy form (Figure 7, reaction 1) [125,126], resulting in sufficient activation energy of high-energy ROOR (80,100 kcal/mol) [127,128] for the formation of ^3^R=O^*^ (74 kcal/mol) (Figure 7, reaction 2). The activation of ROOR to its high-energy form occurs by either thermal or photochemical reactions [125]. It has been widely demonstrated that heat activation of ROOR forms high-energy ROOR, which is known to decompose to two carbonyls, one of which is in the triplet excited state [80,125]. The thermal decomposition of ROOR involves the cleavage of oxygen-oxygen and carbon-carbon bonds [125]. Two distinguishable mechanisms for the thermal decomposition of ROOR have been proposed [127]. The concerted mechanism involves the simultaneous cleavage of oxygen-oxygen and carbon-carbon bonds (Figure 8, reaction 1). The diradical mechanism is a two-step reaction involving the cleavage of bound oxygen, resulting in the formation of diradical, followed by the cleavage, of the carbon-carbon bond (Figure 8, reaction 2) [129]. Alternatively, when ROOR carries an electron-rich group, ^1^R=O^*^and R=O are formed [80,130]. Because the quantum yield of ^1^R=O^*^ formation is one or two orders of magnitude lower than ^3^R=O^*^, the decomposition of ROOR to ^3^R=O^*^ occurs predominantly. Because the activation energy of ^1^R=O^*^ is 80 kcal/mol [131], the decomposition of high energy ROOR to ^1^R=O^*^ is unfeasible from a thermodynamic point of view. The tetroxide formed by the recombination of two ROO^•^ decomposes to ^3^R=O^*^, organic hydroxide (ROH), and molecular oxygen (Figure 6, reaction 2) [132]. The chemistry behind the decomposition of 1,2-dioxetane from a molecular-orbital perspective is discussed more deeply in another review [133]. The decomposition of ROOOOR comprises the transfer of the α-hydrogen of the first ROO^•^ to the second ROO^•^ [114]. The decomposition of the high-energy intermediate ROOOOR is exothermal enough (100–120 kcal/mol) [81] for the formation of ^3^R=O^*^ (74 kcal/mol) (Figure 7, reaction 3). The electronic transition from the triplet excited state to the ground state of carbonyls is associated with photon emission in the near UVA and blue-green regions of the spectrum (350–550 nm) (Figure 7, reaction 4) [134].

#### 3.3.2. Chromophore

In the presence of natural chromophores, such as tetrapyrroles, flavin, pyridine nucleotides, melanin, urocanic acid, and pterins, the energy transfer from ^3^R=O^*^ to chromophore results in the formation of the excited chromophore. Two reaction pathways have been proposed for the energy transfer from ^3^R=O^*^ to the chromophore [135,136,137]. In the direct reaction pathway, the triplet-singlet energy transfer from ^3^R=O^*^ to chromophore forms directly ^1^C^*^. Because the singlet energy level of chromophores (38–52 kcal/mol) is below the triplet energy level of carbonyl (74 kcal/mol), the triplet-singlet energy transfer from ^3^R=O^*^ to chromophore is feasible from a thermodynamic point of view (Figure 7, reaction 5) [138]. Intersystem crossing of ^1^C^*^, comprising changes in the spin orientation results in the formation of ^3^C^*^ (Figure 7, reaction 6). In the induced reaction pathway, the triplet-triplet energy transfer from ^3^R=O^*^ to chromophore forms ^3^C^*^ (Figure 7, reaction 7), which is further transformed to ^1^C^*^ by reverse inter-system crossing (Figure 7, reaction 8). Because the singlet (38–52 kcal/mol) and triplet (28–38 kcal/mol) energy levels of chromophores are below the triplet energy level of carbonyl (74 kcal/mol), the triplet-singlet and triplet-triplet energy transfer from ^3^R=O^*^ to chromophore is feasible from a thermodynamic point of view. The ^1^C^*^ undergoes the electronic transition from the singlet excited state to the ground state, accompanied by photon emission in the green-red region of the spectrum (550–750 nm) (Figure 7, reaction 9). Under anaerobic conditions or in the interior of the membrane, where the concentration of molecular oxygen is low, ^3^C^*^ undergoes the electronic transition from the triplet excited state to the ground state, accompanied by photon emission in the near IR region of the spectrum (750–1000 nm) (Figure 7, reaction 10) [139]. In addition, triplet-singlet energy transfer from ^3^R=O^*^ to melanin is known to be associated with photon emission in near UVA and blue-green regions of the spectrum (360–560 nm) [140,141,142,143].

The photon emission from chlorophyll is in the red region of the spectrum (670–740 nm) [144,145]. The short-wavelength photon emission originates predominantly from free chlorophylls, whereas the long-wavelength photon emission is likely caused by the contribution of chlorophyll coordinated with the protein matrix or reabsorption of photon emission by chlorophylls. The triplet-singlet energy transfer from ^3^R=O^*^ to chlorophylls has been suggested to proceed either via direct or induced reaction pathways [137]. In the case of direct reaction pathways, the triplet-singlet energy transfer from ^3^R=O^*^ to chlorophylls results in the formation of a singlet excited state of chlorophyll. On the contrary, in the induced reaction pathway, the triplet-triplet energy transfer from ^3^R=O^*^ to chlorophylls results in the formation of a triplet excited state of chlorophyll, followed by the transformation to its singlet excited state by reverse inter-system crossing.

#### 3.3.3. Singlet Oxygen

Singlet oxygen is formed by the triplet-singlet energy transfer from ^3^R=O^*^ to molecular oxygen (Figure 7, reaction 12). It was shown that the triplet-singlet energy transfer from ^3^R=O^*^ occurs predominantly to molecular oxygen with the solvent cage [146]. Since the activation energy of ^3^R=O^*^ (74 kcal/mol) is much higher than the activation energy of the first singlet excited state (^1^Δ_g_) (22.5 kcal/mol), the triplet-singlet energy transfer from ^3^R=O^*^ to molecular oxygen is feasible. Alternatively, the ROOOOR decomposes to ^1^O_2_, R=O, and ROH (Figure 7, reaction 11) (Figure 6, reaction 2) [109]. The formation of ^1^O_2_ via the Russell mechanism was reported in chemical systems [108,109,111,114,147,148]. Recently, it has been shown that ^1^O_2_ is formed by the enzymatic (cytochrome c and lactoperoxidase) decomposition of lipid ROOH, followed by the Russell mechanism [149]. The activation energy of ROOOOR (100–120 kcal/mol) is five times higher than the activation energy of the first singlet excited state (^1^Δ_g_), which makes the decomposition of ROOOOR into ^1^O_2_ thermodynamically feasible. In addition, ^1^O_2_ is formed by the triplet-singlet energy transfer from ^3^C^*^ to molecular oxygen (Figure 7, reaction 13). Because the activation energy of several types of chromophore, such as melanin (32 kcal/mol), porphyrin (38 kcal/mol), riboflavin (49.8 kcal/mol), urocanic acid (55 kcal/mol), and pterin (65 kcal/mol), is higher than the activation energy of the first singlet excited state (^1^Δ_g_), the triplet-singlet energy transfer from ^3^C^*^ to molecular oxygen is thermodynamically feasible. The singlet oxygen undergoes an electronic transition from the singlet excited state to the triplet ground state, accompanied by dimol (Figure 7, reaction 14) and monomol (Figure 7, reaction 15) photon emissions in the red (634 and 703 nm) and near IR region (1270 nm) of the spectrum, respectively [81,150].

## 4. Conclusions

In this review, the mechanisms of the formation of electronically excited species during the oxidative metabolic processes are presented. In the initial sections, the formation and the oxidizing property of ROS associated with the formation of electronically excited species are reviewed. This is followed by the detailed mechanistic pathways leading to the formation of electronically excited species, discussed as three distinct phases: 1) The formation of organic radicals, 2) the formation of high-energy intermediates (ROOR or ROOOOR), and 3) the formation of ^3^R=O^*^, ^1^C^*^, ^3^C^*^, and ^1^O_2_. The mechanisms described in this review are mainly based on the experiments performed in chemical systems. In order to understand not just the mechanism but also the probability and reaction yield of the previously described mechanisms, a lot of in vivo experiments have to be done.

## References

[1] Halliwell B., Gutteridge J.M.C. (2007). Free Radical in Biology and Medicine.

[2] Halliwell B. (2011). Free radicals and antioxidants-Quo vadis?. Trends Pharmacol. Sci..

[3] Hawkins C.L., Davies M.J. (2001). Generation and propagation of radical reactions on proteins. Biochim. Biophys. Acta Bioenerget..

[4] Tulah A.S., Birch-Machin M.A. (2013). Stressed out mitochondria: The role of mitochondria in ageing and cancer focussing on strategies and opportunities in human skin. Mitochondrion.

[5] Kroeller-Schoen S., Steven S., Kossmann S., Scholz A., Daub S., Oelze M., Xia N., Hausding M., Mikhed Y., Zinssius E. (2014). Molecular Mechanisms of the Crosstalk Between Mitochondria and NADPH Oxidase Through Reactive Oxygen Species-Studies in White Blood Cells and in Animal Models. Antioxid. Redox Signal..

[6] Radi R. (2013). Peroxynitrite, a Stealthy Biological Oxidant. J. Biol. Chem..

[7] Bartesaghi S., Radi R. (2018). Fundamentals on the biochemistry of peroxynitrite and protein tyrosine nitration. Redox Biol..

[8] Radi R. (2018). Oxygen radicals, nitric oxide, and peroxynitrite: Redox pathways in molecular medicine. Proc. Natl. Acad. Sci. USA.

[9] Gutteridge J.M.C., Halliwell B. (2000). Free radicals and antioxidants in the year 2000—A historical look to the future. Ann. N. Y. Acad. Sci..

[10] Halliwell B., Gutteridge J.M.C. (1995). The definition and measurement of antioxidants in biological systems. Free Radic. Biol. Med..

[11] Hellemans L., Corstjens H., Neven A., Declercq L., Maes D. (2003). Antioxidant enzyme activity in human stratum corneum shows seasonal variation with an age-dependent recovery. J. Investig. Dermatol..

[12] Auf Dem Keller U., Kumin A., Braun S., Werner S. (2006). Reactive oxygen species and their detoxification in healing skin wounds. J. Investig. Dermatol. Symp. Proc..

[13] Darvin M.E., Patzelt A., Knorr F., Blume-Peytavi U., Sterry W., Lademann J. (2008). One-year study on the variation of carotenoid antioxidant substances in living human skin: Influence of dietary supplementation and stress factors. J. Biomed. Opt..

[14] Tiwari S., Mishra P.C. (2011). Urocanic acid as an efficient hydroxyl radical scavenger: A quantum theoretical study. J. Mol. Model..

[15] Linton S., Davies M.J., Dean R.T. (2001). Protein oxidation and ageing. Exp. Gerontol..

[16] Celaje J.A., Zhang D., Guerrero A.M., Selke M. (2011). Chemistry of trans-Resveratrol with Singlet Oxygen: 2+2 Addition, 4+2 Addition, and Formation of the Phytoalexin Moracin M. Org. Lett..

[17] Jacobson M.D. (1996). Reactive oxygen species and programmed cell death. Trends Biochem. Sci..

[18] Bennett M.F., Robinson M.K., Baron E.D., Cooper K.D. (2008). Skin immune systems and inflammation: Protector of the skin or promoter of aging?. J. Investig. Dermatol. Symp. Proc..

[19] Cilento G., Adam W. (1995). From free radicals to electronically excited species. Free Radic. Biol. Med..

[20] Mano C.M., Prado F.M., Massari J., Ronsein G.E., Martinez G.R., Miyamoto S., Cadet J., Sies H., Medeiros M.H.G., Bechara E.J.H. (2014). Excited singlet molecular O-2 ((1)Delta g) is generated enzymatically from excited carbonyls in the dark. Sci. Rep..

[21] Cifra M., Pospíšil P. (2014). Ultra-weak photon emission from biological samples: Definition, mechanisms, properties, detection and applications. J. Photochem. Photobiol. B Biol..

[22] Prasad A., Pospisil P. (2013). Towards the two-dimensional imaging of spontaneous ultra-weak photon emission from microbial, plant and animal cells. Sci. Rep..

[23] Madl P., Verwanger T., Geppert M., Scholkmann F. (2017). Oscillations of ultra-weak photon emission from cancer and non-cancer cells stressed by culture medium change and TNF-alpha. Sci. Rep..

[24] Rac M., Sedlarova M., Pospisil P. (2015). The formation of electronically excited species in the human multiple myeloma cell suspension. Sci. Rep..

[25] He M., van Wijk E., van Wietmarschen H., Wang M., Sun M.M., Koval S., van Wijk R., Hankemeier T., van der Greef J. (2017). Spontaneous ultra-weak photon emission in correlation to inflammatory metabolism and oxidative stress in a mouse model of collagen-induced arthritis. J. Photochem. Photobiol. B.

[26] Prasad A., Ferretti U., Sedlarova M., Pospisil P. (2016). Singlet oxygen production in Chlamydomonas reinhardtii under heat stress. Sci. Rep..

[27] Usui S., Tada M., Kobayashi M. (2019). Non-invasive visualization of physiological changes of insects during metamorphosis based on biophoton emission imaging. Sci. Rep..

[28] Van Wijk R., Van Wijk E. (2005). Ultraweak Photon Emission from Human Body.

[29] Biesalski H.K., Obermueller-Jevic U.C. (2001). UV light, beta-carotene and human skin-Beneficial and potentially harmful effects. Arch. Biochem. Biophys..

[30] Hao O.Y., Stamatas G., Saliou C., Kollias N. (2004). A chemiluminescence study of UVA-induced oxidative stress in human skin in vivo. J. Investig. Dermatol..

[31] Rattan S.I.S., Fernandes R.A., Demirovic D., Dymek B., Lima C.F. (2009). Heat stress and hormetin-induced hormesis in human cells: Effects on aging, wound healing, angiogenesis, and differentiation. Dose-response.

[32] Kobayashi M., Kikuchi D., Okamura H. (2009). Imaging of Ultraweak Spontaneous Photon Emission from Human Body Displaying Diurnal Rhythm. PLoS ONE.

[33] Prasad A., Pospisil P. (2011). Linoleic Acid-Induced Ultra-Weak Photon Emission from Chlamydomonas reinhardtii as a Tool for Monitoring of Lipid Peroxidation in the Cell Membranes. PLoS ONE.

[34] Prasad A., Pospisil P. (2011). Two-dimensional imaging of spontaneous ultra-weak photon emission from the human skin: Role of reactive oxygen species. J. Biophotonics.

[35] Rastogi A., Pospíšil P. (2012). Production of hydrogen peroxide and hydroxyl radical in potato tuber during the necrotrophic phase of hemibiotrophic pathogen Phytophthora infestans infection. J. Photochem. Photobiol. B Biol..

[36] Burgos R.C.R., Schoeman J.C., van Winden L.J., Cervinkova K., Ramautar R., Van Wijk E.P.A., Cifra M., Berger R., Hankemeier T., van der Greef J. (2017). Ultra-weak photon emission as a dynamic tool for monitoring oxidative stress metabolism. Sci. Rep..

[37] Kamal A.M., Komatsu S. (2016). Proteins involved in biophoton emission and flooding-stress responses in soybean under light and dark conditions. Mol. Biol. Rep..

[38] Cadenas E., Arad I.D., Boveris A., Fisher A.B., Chance B. (1980). Partial Spectral-Analysis of the Hydroperoxide-Induced Chemi-Luminescence of the Perfused Lung. FEBS Lett..

[39] Van Wijk R., Ackerman J.M., Van Wijk E.P.A. (2005). Color filters and human photon emission: Implications for auriculomedicine. Explor. J. Sci. Heal..

[40] Cadenas E., Wefers H., Sies H. (1981). Low-Level Chemi-Luminescence of Isolated Hepatocytes. Eur. J. Biochem..

[41] Mathew B.G., Roy D. (1992). Weak luminescence from the frozen-thawed root tips of *Cicer arietinum* L.. J. Photochem. Photobiol. B Biol..

[42] Pospíšil P., Prasad A., Rác M. (2014). Role of reactive oxygen species in ultra-weak photon emission in biological systems. J. Photochem. Photobiol. B Biol..

[43] Han D., Williams E., Cadenas E. (2001). Mitochondrial respiratory chain-dependent generation of superoxide anion and its release into the intermembrane space. Biochem. J..

[44] Auchere F., Rusnak F. (2002). What is the ultimate fate of superoxide anion in vivo?. J. Biol. Inorg. Chem..

[45] Pospisil P. (2012). Molecular mechanisms of production and scavenging of reactive oxygen species by photosystem II. Biochim. Biophys. Acta Bioenerget..

[46] Asada K. (2006). Production and scavenging of reactive oxygen species in chloroplasts and their functions. Plant Physiol..

[47] Barber M.J., Kay C.J. (1996). Superoxide production during reduction of molecular oxygen by assimilatory nitrate reductase. Arch. Biochem. Biophys..

[48] Lambert A.J., Brand M.D. (2004). Superoxide production by NADH: Ubiquinone oxidoreductase (complex I) depends on the pH gradient across the mitochondrial inner membrane. Biochem. J..

[49] Wood P.M. (1988). The potential diagram for oxygen at PH-7. Biochem. J..

[50] Aikens J., Dix T.A. (1991). Perhydroxyl radical (HOO^.^) initiated lipid-peroxidation-the role of fatty acid hydroperoxide. J. Biol. Chem..

[51] Gebicki J.M., Bielski B.H.J. (1981). Comparison of the capacities of the perhydroxyl and the speroxide radicals to initiate chain oxidation of linoleic acid. J. Am. Chem. Soc..

[52] Winterbourn C.C., Kettle A.J. (2003). Radical-radical reactions of superoxide: A potential route to toxicity. Biochem. Biophys. Res. Commun..

[53] Barbouti A., Doulias P.T., Zhu B.Z., Frei B., Galaris D. (2001). Intracellular iron, but not copper, plays a critical role in hydrogen peroxide-induced DNA damage. Free Radical Biol. Med..

[54] Dahlgren C., Karlsson A., Bylund J. (2007). Measurement of respiratory burst products generated by professional phagocytes. Methods Mol. Biol..

[55] Daithankar V.N., Wang W., Trujillo J.R., Thorpe C. (2012). Flavin-linked Erv-family sulfhydryl oxidases release superoxide anion during catalytic turnover. BioChemistry.

[56] Wood P.M. (1987). The 2 Redox Potentials for Oxygen Reduction to Superoxide. Trends Biochem. Sci..

[57] Culotta V.C., Yang M., O’Halloran T.V. (2006). Activation of superoxide dismutases: Putting the metal to the pedal. Biochim. Biophysica Acta-Mol. Cell Res..

[58] Halliwell B., Clement M.V., Long L.H. (2000). Hydrogen peroxide in the human body. FEBS Lett..

[59] Kale R., Hebert A.E., Frankel L.K., Sallans L., Bricker T.M., Pospíšil P. (2017). Amino acid oxidation of the D1 and D2 proteins by oxygen radicals during photoinhibition of Photosystem II. Proc. Natl. Acad. Sci. USA.

[60] Hoffmann M.E., Meneghini R. (1979). Action of hydrogen peroxide on human fibroblast in culture. PhotoChem. Photobiol..

[61] Stadtman E.R., Levine R.L. (2003). Free radical-mediated oxidation of free amino acids and amino acid residues in proteins. Amino Acids.

[62] Kim Y.H., Berry A.H., Spencer D.S., Stites W.E. (2001). Comparing the effect on protein stability of methionine oxidation versus mutagenesis: Steps toward engineering oxidative resistance in proteins. Protein Eng..

[63] Ashby M.T., Nagy P. (2006). On the kinetics and mechanism of the reaction of cysteine and hydrogen peroxide in aqueous solution-Commentary. J. Pharm. Sci..

[64] Winterbourn C.C. (1995). Toxicity of iron and hydrogen peroxide: The Fenton reaction. Toxicol. Lett..

[65] Prousek J. (2007). Fenton chemistry in biology and medicine. Pure Appl. Chem..

[66] Buettner G.R. (1993). The pecking order of free-radicals and antioxidants-lipid-peroxidation, alpha-tocopherol, and ascorbate. Arch. BioChem. Biophys..

[67] Pierre J.L., Fontecave M. (1999). Iron and activated oxygen species in biology: The basic chemistry. Biometals.

[68] Bresgen N., Jaksch H., Lacher H., Ohlenschlager I., Uchida K., Eckl P.M. (2010). Iron-mediated oxidative stress plays an essential role in ferritin-induced cell death. Free Radic. Biol. Med..

[69] Waldo G.S., Wright E., Whang Z.H., Briat J.F., Theil E.C., Sayers D.E. (1995). Formation of the ferritin iron mineral occurs in plastids-an X-ray-absorption spectroscopy study. Plant Physiol..

[70] Koppenol W.H., Butler J., Vanleeuwen J.W. (1978). Haber-Weiss Cycle. PhotoChem. Photobiol..

[71] Pospíšil P. (2009). Production of reactive oxygen species by photosystem II. Biochim. Biophysica Acta-Bioenerget..

[72] Stadtman E.R. (1993). Oxidation of free amino-acids and amino-acid-residues in proteins by radiolysis and by meral-catalyzed reactions. Annu. Rev. BioChem..

[73] Aust A.E., Eveleigh J.F. (1999). Mechanisms of DNA oxidation. Proc. Soc. Exp. Biol. Med..

[74] Balasubramanian B., Pogozelski W.K., Tullius T.D. (1998). DNA strand breaking by the hydroxyl radical is governed by the accessible surface areas of the hydrogen atoms of the DNA backbone. Proc. Natl. Acad. Sci. USA.

[75] Pogozelski W.K., Tullius T.D. (1998). Oxidative strand scission of nucleic acids: Routes initiated by hydrogen abstraction from the sugar moiety. Chem. Rev..

[76] Henle E.S., Linn S. (1997). Formation, prevention, and repair of DNA damage by iron hydrogen peroxide. J. Biol. Chem..

[77] Halliwell B., Aruoma O.I. (1991). DNA damage by oxygen-derived species-its mechanism and measurement in mammalian systems. FEBS Lett..

[78] Ogilby P.R. (2010). Singlet oxygen: There is indeed something new under the sun. Chem. Soc. Rev..

[79] DeRosa M.C., Crutchley R.J. (2002). Photosensitized singlet oxygen and its applications. Coord. Chem. Rev..

[80] Adam W., Cilento G. (1982). Chemical and Biology Generation of Excited States.

[81] Adam W., Kazakov D.V., Kazakov V.P. (2005). Singlet-oxygen chemiluminescence in peroxide reactions. Chem. Rev..

[82] Stratton S.P., Liebler D.C. (1997). Determination of singlet oxygen-specific versus radical-mediated lipid peroxidation in photosensitized oxidation of lipid bilayers: Effect of beta-carotene and alpha-tocopherol. BioChemistry.

[83] Gracanin M., Hawkins C.L., Pattison D.I., Davies M.J. (2009). Singlet-oxygen-mediated amino acid and protein oxidation: Formation of tryptophan peroxides and decomposition products. Free Radic. Biol. Med..

[84] Pryor W.A., Stanley J.P., Blair E., Cullen G.B. (1976). Autoxidation of polyunsaturated fatty-acids. 1. effect of ozone on autoxidation of neat methyl linoleate and methyl linolenate. Arch. Environ. Health.

[85] D’Ambrosio P., Tonucci L., d’Alessandro N., Morvillo A., Sortino S., Bressan M. (2011). Water-Soluble Transition-Metal-Phthalocyanines as Singlet Oxygen Photosensitizers in Ene Reactions. Eur. J. Inorg. Chem..

[86] Griesbeck A.G., de Kiff A. (2013). A New Directing Mode for Singlet Oxygen Ene Reactions: The Vinylogous Gem Effect Enables a O-1(2) Domino Ene/ 4+2 Process. Org. Lett..

[87] Ravanat J.L., Di Mascio P., Martinez G.R., Medeiros M.H.G., Cadet J. (2000). Singlet oxygen induces oxidation of cellular DNA. J. Biol. Chem..

[88] Tsunoda H., Kudo T., Masaki Y., Ohkubo A., Seio K., Sekine M. (2011). Biochemistry behavior of N-oxidized cytosine and adenine bases in DNA polymerase-mediated primer extension reactions. Nucleic Acids Res..

[89] Nguyen K.V., Muller J.G., Burrows C.J. (2011). Oxidation of 9-beta-D-ribofuranosyl uric acid by one-electron oxidants versus singlet oxygen and its implications for the oxidation of 8-oxo-7,8-dihydroguanosine. Tetrahedron Lett..

[90] Kang P., Foote C.S. (2002). Formation of transient intermediates in low-temperature photosensitized oxidation of an 8-C-13-guanosine derivative. J. Am. Chem. Soc..

[91] Prat F., Houk K.N., Foote C.S. (1998). Effect of guanine stacking on the oxidation of 8-oxoguanine in B-DNA. J. Am. Chem. Soc..

[92] Hatz S., Poulsen L., Ogilby P.R. (2008). Time-resolved singlet oxygen phosphorescence measurements from photosensitized experiments in single cells: Effects of oxygen diffusion and oxygen concentration. PhotoChem. Photobiol..

[93] Da Silva E.F.F., Pedersen B.W., Breitenbach T., Toftegaard R., Kuimova M.K., Arnaut L.G., Ogilby P.R. (2012). Irradiation-and Sensitizer-Dependent Changes in the Lifetime of Intracellular Singlet Oxygen Produced in a Photosensitized Process. J. Phys. Chem. B.

[94] Breitenbach T., Kuimova M.K., Gbur P., Hatz S., Schack N.B., Pedersen B.W., Lambert J.D.C., Poulsen L., Ogilby P.R. (2009). Photosensitized production of singlet oxygen: Spatially-resolved optical studies in single cells. Photochem. PhotoBiol. Sci..

[95] Di Mascio P., Martinez G.R., Miyamoto S., Ronsein G.E., Medeiros M.H.G., Cadet J. (2019). Singlet Molecular Oxygen Reactions with Nucleic Acids, Lipids, and Proteins. Chem. Rev..

[96] Girotti A.W. (1998). Lipid hydroperoxide generation, turnover, and effector action in biological systems. J. Lipid Res..

[97] Leach A.G., Houk K.N., Foote C.S. (2008). Theoretical Prediction of a Perepoxide Intermediate for the Reaction of Singlet Oxygen with trans-Cyclooctene Contrasts with the Two-Step No-Intermediate Ene Reaction for Acyclic Alkenes. J. Org. Chem..

[98] Dean R.T., Gieseg S., Davies M.J. (1993). Reactive Species and Their Accumulation on Radical-Damaged Proteins. Trends Biochem. Sci..

[99] Simpson J.A., Narita S., Gieseg S., Gebicki S., Gebicki J.M., Dean R.T. (1992). Long-lived reactive species on free-radical-damaged proteins. Biochem. J..

[100] Greer A., Vassilikogiannakis G., Lee K.C., Koffas T.S., Nahm K., Foote C.S. (2000). Reaction of singlet oxygen with trans-4-propenylanisole. Formation of 2+2 products with added acid. J. Org. Chem..

[101] Fedorova G.F., Trofimov A.V., Vasil’ev R.F., Veprintsev T.L. (2007). Peroxy-radical-mediated chemiluminescence: Mechanistic diversity and fundamentals for antioxidant assay. Arkivoc.

[102] Uemi M., Ronsein G.E., Miyamoto S., Medeiros M.H.G., Di Mascio P. (2009). Generation of Cholesterol Carboxyaldehyde by the Reaction of Singlet Molecular Oxygen O-2 ((1)Delta(g)) as Well as Ozone with Cholesterol. Chem. Res. Toxicol..

[103] Clennan E.L. (2000). New mechanistic and synthetic aspects of singlet oxygen chemistry. Tetrahedron.

[104] Adam W., Bosio S.G., Turro N.J. (2002). Highly diastereoselective dioxetane formation in the photooxygenation of enecarbamates with an oxazolidinone chiral auxiliary: Steric control in the 2+2 cycloaddition of singlet oxygen through conformational alignment. J. Am. Chem. Soc..

[105] Clennan E.L., Pace A. (2005). Advances in singlet oxygen chemistry. Tetrahedron.

[106] Corey E.J., Wang Z. (1994). Conversion of arachidonic acid to the prostaglandin endoperoxide PGG(2), a chemical analog of the biosynthetic-pathway. Tetrahedron Lett..

[107] Miyamoto S., Rettori D., Augusto O., Martinez G.R., Medeiros M.H.G., Di Mascio P. (2004). Linoleic acid hydroperoxide reacts with hypochlorite generating peroxyl radical intermediates and singlet oxygen. Free Radic. Biol. Med..

[108] Prado F.M., Oliveira M.C.B., Miyamoto S., Martinez G.R., Medeiros M.H.G., Ronsein G.E., Di Mascio P. (2009). Thymine hydroperoxide as a potential source of singlet molecular oxygen in DNA. Free Radic. Biol. Med..

[109] Russell G.A. (1957). Deuterium-isotope Effects in the Autoxidation of Aralkyl Hydrocarbons. Mechanism of the Interaction of Peroxy Radicals. J. Am. Chem. Soc..

[110] Miyamoto S., Martinez G.R., Medeiros M.H.G., Di Mascio P. (2014). Singlet molecular oxygen generated by biological hydroperoxides. J. PhotoChem. Photobiol. B Biol..

[111] Miyamoto S., Di Mascio P., Kato Y. (2014). Lipid Hydroperoxides as a Source of Singlet Molecular Oxygen. Lipid Hydroperoxide-Derived Modification of Biomolecules.

[112] Cadenas E., Sies H. (2000). Formation of electronically excited states during the oxidation of arachidonic acid by prostaglandin endoperoxide synthase. Methods in Enzymology.

[113] Hall R., Chamulitrat W., Takahashi N., Chignell C., Mason R. (1989). Detection of Singlet oxygem phosphorescence during chloroperoxidase-catalyzed decomposition of ethyl hydroperoxide. J. Biol. Chem..

[114] Howard J.A., Ingold K.U. (1967). Absolute rate constants for hydrocarbon autooxidation.V. Hydroperoxy radical in chain propagation and termination. Can. J. Chem..

[115] Cilento G., Nascimento A. (1993). Generation of electronically excited triplet species at the cellular level: A potential source of genotoxicity. Toxicol. Lett..

[116] Boveris A., Cadenas E., Reiter R., Filipkowski M., Nakase Y., Chance B. (1980). Organ Chemi-Luminescence-Non-Invasive Assay for Oxidative Radical Reactions. Proc. Natl. Acad. Sci. USA.

[117] Bennett M., Mehta M., Grant M. (2005). Biophoton imaging: A nondestructive method for assaying R gene responses. Mol. Plant-Microbe Interact..

[118] Darmanyan A.P., Foote C.S. (1993). Solvent effects on singlet oxygen yield from N,PI-asterisk and PI,PI-asterisk triplet carbonyl compounds. J. Phys. Chem..

[119] Mansfield J.W. (2005). Biophoton distress flares signal the onset of the hypersensitive reaction. Trends Plant Sci..

[120] Niu E.P., Mau A.W.H., Ghiggino K.P. (1991). Dye-Sensitized Photooxidation of Anthracene and Its Derivatives in Nafion Membrane. Aust. J. Chem..

[121] Cilento G., Adam W. (1988). Photochemistry and Photobiology without Light. PhotoChem. Photobiol..

[122] Kopecky K.R., Mumford C. (1969). Luminescence in thermal decomposition of 3,3,4-trimethyl-1,2-dioxetane. Can. J. Chem..

[123] Cilento G., Debaptista C., Brunetti I.L. (1994). Triplet carbonyls: From photophysics to biochemistry. J. Mol. Struct..

[124] Kopecky K.R., Filby J.E. (1979). Yields of excited-states from thermolysis of some 1,2-dioxetanes. Can. J. Chem..

[125] Turro N.J., Lechtken P. (1973). Biacetyl sensitized decomposition of tetramethyl-1,2-dioxetane-example of anti-Stokes sensitization involving a masked excited state. Tetrahedron Lett..

[126] Matsumoto M. (2004). Advanced chemistry of dioxetane-based chemiluminescent substrates originating from bioluminescence. J. Photochem. Photobiol. C.

[127] Richardson W.H., Anderegg J.H., Price M.E., Tappen W.A., Oneal H.E. (1978). Kinetics and mechanisms of thermal decomposition of triphenyl-1,1,2-dioxetane. J. Org. Chem..

[128] Akasaka T., Fukuoka K., Ando W. (1989). A 3-methylene-1,2-dioxetane as a possible chemiluminescent intermediate in singlet oxygenation of allene. Bull. Chem. Soc. Jpn..

[129] Baumstark A.L., Anderson S.L., Sapp C.J., Vasquez P.C. (2001). Thermolysis of 3-alkyl-3-methyl-1,2-dioxetanes: Activation parameters and chemiexcitation yields. Heteroat. Chem..

[130] Nery A.L.P., Weiss D., Catalani L.H., Baader W.J. (2000). Studies on the intramolecular electron transfer catalyzed thermolysis of 1,2-dioxetanes. Tetrahedron.

[131] Carey F., Sundberg R. (1984). Advanced Organic Chemisty Part A Structure and Mechanisms.

[132] Miyamoto S., Ronsein G.E., Prado F.M., Uemi M., Correa T.C., Toma I.N., Bertolucci A., Oliveira M.C.B., Motta F.D., Medeiros M.H.G. (2007). Biological hydroperoxides and singlet molecular oxygen generation. IUBMB Life.

[133] Vacher M., Galvan I.F., Ding B.W., Schramm S., Berraud-Pache R., Naumov P., Ferre N., Liu Y.J., Navizet I., Roca-Sanjuan D. (2018). Chemi-and Bioluminescence of Cyclic Peroxides. Chem. Rev..

[134] Augusto O., Cilento G. (1979). Dark Excitation of Chlorophyll. PhotoChem. Photobiol..

[135] Bohne C., Campa A., Cilento G., Nassi L., Villablanca M. (1986). Chlorophyll-an efficient detector of electronically excited species in *Biochem.* systems. Anal. BioChem..

[136] Campa A., Nassi L., Cilento G. (1984). Triplet Energy-Transfer to Chloroplasts from Peroxidase-Generated Excited Aliphatic-Aldehydes. PhotoChem. Photobiol..

[137] Marder J.B., Droppa M., Caspi V., Raskin V.I., Horvath G. (1998). Light-independent thermoluminescence from thylakoids of greening barley leaves. Evidence for involvement of oxygen radicals and free chlorophyll. Physiol. Plant..

[138] Porcal G., Bertolotti S.G., Previtah C.M., Encinas M.V. (2003). Electron transfer quenching of singlet and triplet excited states of flavins and lumichrome by aromatic and aliphatic electron donors. Phys. Chem. Chem. Phys..

[139] Krasnovsky A.A., Neverov K.V., Egorov S.Y., Roeder B., Levald T. (1990). Photophysical Studies of Pheophorbide-a and Pheophytin-a Phosphorescence and Photo-Sensitized Singlet Oxygen Luminescence. J. Photochem. Photobiol. B.

[140] Ortega-Ojeda F., Calcerrada M., Ferrero A., Campos J., Garcia-Ruiz C. (2018). Measuring the Human Ultra-Weak Photon Emission Distribution Using an Electron-Multiplying, Charge-Coupled Device as a Sensor. Sensors.

[141] Tsuchida K., Iwasa T., Kobayashi M. (2018). Noninvasive imaging of UV-induced oxidative stress in human skin using ultra-weak photon emission. J. Investig. Dermatol..

[142] Kobayashi M., Iwasa T., Tada M. (2016). Polychromatic spectral pattern analysis of ultra-weak photon emissions from a human body. J. Photochem. Photobiol. B.

[143] Prasad A., Balukova A., Pospisil P. (2018). Triplet Excited Carbonyls and Singlet Oxygen Formation During Oxidative Radical Reaction in Skin. Front. Physiol..

[144] Hideg E., Inaba H. (1991). Biophoton Emission (Ultraweak Photoemission) from Dark Adapted Spinach-Chloroplasts. PhotoChem. Photobiol..

[145] Kalaji H.M., Goltsev V., Bosa K., Allakhverdiev S.I., Strasser R.J., Govindjee (2012). Experimental in vivo measurements of light emission in plants: A perspective dedicated to David Walker. Photosynth. Res..

[146] Kellogg R.E. (1969). Mechanism of chemiluminescence from peroxy radicals. J. Am. Chem. Soc..

[147] Niu Q.J., Mendenhall G.D. (1992). Yields of singlet molecular oxygen from peroxyl radical termination. J. Am. Chem. Soc..

[148] Kanofsky J.R. (1989). Singlet oxygen production from the peroxidase catalyzed formation of styrene glutathione adducts. Biochem. Biophys. Res. Commun..

[149] Sun S., Bao Z., Ma H., Zhang D., Zheng X. (2007). Singlet oxygen generation from the decomposition of alpha-linolenic acid hydroperoxide by cytochrome c and lactoperoxidase. BioChemstry.

[150] Koh E., Fluhr R. (2016). Singlet oxygen detection in biological systems: Uses and limitations. Plant Signal. Behav..

